# Mineralocorticoid effects in the late gestation ovine fetal lung

**DOI:** 10.14814/phy2.12066

**Published:** 2014-07-17

**Authors:** Jarret McCartney, Elaine M. Richards, Charles E. Wood, Maureen Keller‐Wood

**Affiliations:** 1 Department of Pharmacodynamics, University of Florida, Gainesville, Florida, USA; 2 Department of Physiology and Functional Genomics, University of Florida, Gainesville, Florida, USA

**Keywords:** Fetus, glucocorticoid, lung, mineralocorticoid

## Abstract

This study was designed to determine the effects of corticosteroids at MR in the late‐gestation fetal lung. Since both the mineralocorticoid receptor (MR) and the glucocorticoid receptor (GR) are expressed at relatively high levels in the fetal lung, endogenous corticosteroids may act at MR as well as GR in the preterm fetal lung. The GR agonist, betamethasone, the MR agonist, aldosterone, or both were infused intravenously for 48 h in ovine fetuses of approximately 130 days gestation. Effects on airway pressures during stepwise inflation of the in situ lung, expression of ENaC alpha (SCNN1A), ENaC beta (SCNN1B), and Na,K ATPase (ATP1A1), and elastin and collagen content were determined after the infusions. We found that aldosterone significantly reduced the airway pressure measured during the initial step in inflation of the lung, although aldosterone had no overall effect on lung compliance, nor did aldosterone induce expression of ENaC*α*, ENaC*β* or Na,K ATPase*α*1. Betamethasone significantly increased expression of the epithelial sodium channel (ENaC) subunit mRNAs, and collagen and elastin content in the lungs, although this dose of betamethasone also had no effect on lung compliance. There was no synergy between effects of the MR and GR agonists. Transcriptomic analysis suggested that although aldosterone did not alter genes in pathways related to epithelial sodium transport, aldosterone did alter genes in pathways involved in cell proliferation in the lungs. The results are consistent with corticosteroid‐induced fluid reabsorption at birth through GR rather than MR, but suggest that MR facilitates lung maturation, and may contribute to inflation with the first breaths via mechanisms distinct from known aldosterone effects in other epithelia.

## Abstract (precis)

Infusion of the mineralocorticoid receptor agonist, aldosterone, to the ovine fetus resulted in reduced airway pressures with initial lung inflation. However, aldosterone did not alter lung surfactant or epithelial sodium transport genes which are classical MR gene targets. Transcriptomic analysis revealed an aldosterone effect on genes related to cell cycle, suggesting that MR have a role distinct form that of GR in the maturing lung.

## Introduction

Fetal plasma cortisol secretion increases exponentially in both sheep and primates before parturition (Bassett and Thorburn 1969) facilitating maturation of fetal lungs, liver, kidney, and brain (Liggins 1994). Preterm infants delivered prematurely and/or by cesarean section before the prepartum increase in cortisol are at a greater risk of developing transient tachypnea of the newborn (Ramachandrappa and Jain 2008). Antenatal administration of synthetic glucocorticoids such as dexamethasone and betamethasone stimulates fetal lung maturation, increases lung viability, and decreases neonatal morbidity/mortality (Ballard and Ballard 1996). Effective lung function at birth requires reabsorption of lung liquid via mechanisms involving epithelial sodium and chloride transport (reviewed in Jain and Eaton 2006). Glucocorticoid action contributes to normal lung liquid resorption at birth through the increasing expression of proteins in the maturing lung, including surfactant proteins, antioxidants, the *β*‐adrenergic receptor, and both Na,K ATPase and the epithelial sodium channel, ENaC (reviewed in Ballard and Ballard 1995).

The maturing ovine lung expresses mineralocorticoid receptors (MR) as well as glucocorticoid receptors (GR) and predominately expresses 11*β*‐hydroxysteroid dehydrogenase1 with little 11*β*‐hydroxysteroid dehydrogenase activity (Wood and Srun 1995; Keller‐Wood et al. 2009). Although in rodents MR is expressed as only near term or postnatally (Rosenfeld et al. 1988; Brown et al. 1996; Diaz et al. 1998), in humans MR have been found to be expressed as early as 12 weeks of gestation (Condon et al. 1998; Hirasawa et al. 1999) and activity of 11*β*‐hydroxysteroid dehydrogenase1 predominates (Murphy 1981; Yang et al. 2009), suggesting that circulating cortisol would bind at high‐affinity MR well before the prepartum cortisol surge. Thus, it is likely that endogenous increases in cortisol act at MR as well as at GR in the fetal lung. However, although MR and GR share the same DNA response element, transcriptional activity of the receptors differs (Rupprecht et al. 1993; Kolla et al. 1999). In the postnatal kidney, MR are known to effect sodium reabsorption through induction of SGK, ENaC*α,* and Na,K ATPase (Chen et al. 1999; Pearce et al. 2000), and it is known that absence of ENaC‐*α* in the lung alters lung liquid resorption at birth (Hummler et al. 1996). Although it has been proposed that MR may have developmental effects in several tissues (reviewed in Martinerie et al. 2013), effects of MR in the fetal lung are less clear. Although aldosterone has effects on sodium conductance (Champigny et al. 1994), blockade or knockout of MR does not appear to alter transcription of the genes related to channel proteins (Berger et al. 2000; Keller‐Wood et al. 2011).

These experiments test the hypothesis that MR elicits effects on the preterm lung. As aldosterone had been shown to cause lung liquid reabsorption in the fetal guinea pig (Kindler et al. 1993) and influences lung edema clearance and Na,K ATPase activity in adult rats (Olivera et al. 2000; Suzuki et al. 2001), and we have previously found an effect of mineralocorticoid blockade on lung liquid composition (Keller‐Wood et al. 2011), we tested the following hypotheses: (i) that aldosterone action at MR in the prenatal lung would induce similar gene expression as that in the postnatal kidney, that is, SGK, ENaC*α* and Na, K ATPase*α*1, (ii) that aldosterone would alter lung compliance, (iii) that aldosterone and betamethasone can synergize to induce ENaC and Na, K ATPase subunit expression. Although there are known effects of high‐dose glucocorticoid therapy on these genes (Venkatesh et al. 1993; Tchepichev et al. 1995; Venkatesh and Katzberg 1997; Itani et al. 2002; Nakamura et al. 2002), and on lung compliance and respiratory function at birth (Ikegami et al. 1997; McEvoy et al. 2000), the study was designed to test whether the combined infusion of submaximal doses of the MR agonist, aldosterone, and submaximal doses of the GR agonist, betamethasone, would produce a synergist effect on gene expression or lung compliance.

## Methods

### Experiment procedure

All animal use was approved by the UF Institutional Animal Care and Use Committee. Catheters were placed in 22 ewes of mixed‐Western breed and their fetuses (17 twin and five singleton pregnancies at 122–124 days gestation; term of 145 days) (Reini et al. 2008). Ewes were anesthetized with isoflurane (2–3%) before and during surgery, and catheters were placed in both fetal tibial arteries, saphenous veins, and in the amniotic space; maternal femoral artery and vein catheters were also placed. The catheters were routed to an exit site on the ewe's flank and secured in a pouch. All ewes were treated with flunixin meglumine (1 mg kg^−1^ IM; Fort Dodge Animal Health, Fort Dodge, IA) before recovery from anesthesia; animals were treated with a second dose on the next morning. Polyflex (500 mg SC bid; Fort Dodge Animal Health) was administered for 3 days postoperatively and body temperature was monitored for 5 days. Ewes were given 2.5 kg of ruminant lab diet each day; daily food intake was monitored on each postoperative day.

At least 5 days after surgery, fetuses were treated for 48 h with MR agonist, aldosterone (Aldo; 0.2 mg; *n* = 5, all twin pregnancies), GR agonist (betamethasone; 0.25 mg or 0.75 mg *n* = 2 twin and 2 singleton fetuses at each dose), or 0.2 mg aldosterone combined with either betamethasone dose (Aldo/0.25Beta *n* = 4 twin fetuses, and Aldo/0.75Beta; *n *= 3 twin and 1 singleton fetus). In twin pregnancies, one fetus received corticosteroids and the second uninfused fetus served as control (*n* = 9 after exclusion of seven hypoxic twins with *P*_O2 _< 17: one twin with a malfunctioning stopcock during lung inflations was not used for lung compliance measures); one singleton pregnancy was also used as a control. Administration of 0.75 mg betamethasone consisted of a 0.25 mg bolus followed by infusion of 0.50 mg/48 h. The steroids were delivered as intravenous infusions of aldosterone hemisuccinate or betamethasone disodium phosphate (Steraloids, Newport, RI) in saline at 1.45 mL h^−1^. While it is a standard clinical practice to administer single or multiple doses of betamethasone, we infused agonists in our animal model to produce steady‐state levels. These steroid infusion rates produced much lower effective steroid concentrations than those used clinically or shown to be effective at increasing lung compliance in the fetal lamb after fetal intramuscular injection (Moss et al. 2003). The doses used in our study were chosen to model the effect at MR and at GR of the increases in the endogenous cortisol in the days before the final dramatic increase in cortisol at term to model steroid action in the near‐term fetus. The doses were based on relative affinity of the agonists at MR and GR, predicted agonist clearance rates, and estimated fetal body weight. Our initial dose of 0.25 mg betamethasone over 48 h was chosen to produce similar effects to the infusion of 2 μg min^−1^ cortisol, which had been used in earlier studies in the laboratory (Wood 1986) and based on the relative efficacy of betamethasone of 25‐fold relative to cortisol. We subsequently predicted that the betamethasone dose of 0.25 m/48 h would produce a concentration of approximately 1 nmol/L^−1^ free steroid based on the clearance calculated from peak concentration and area under the curve data after injection in the fetal sheep (Moss et al. 2003). This concentration exceeds the EC50 for betamethasone at the human GR, but would produce submaximal GR responses (Grossmann et al. 2004). The higher dose of betamethasone used a loading dose to more quickly increase plasma betamethasone concentrations, followed by a higher infusion rate of the steroid. The betamethasone doses should have little or no effect at MR. In contrast, the plasma concentrations produced by this dose of aldosterone are predicted to fully occupy and activate the MR (Richards et al. 2003). Throughout the study, ewes remained in their pens (approximately 25 square feet) and the infusions were performed without restraint of the ewes in order to minimize effects on maternal and fetal cortisol concentrations. The infusions were delivered by syringe pump (Razel Scientific Instruments, St. Albans, VT) through a sterile filter (0.2 micron; EMD Millipore, Billerica, MA); the pump was placed above the pen, and the fetal catheter was routed to the pump through a flexible duct connected to a swivel as to allow a free movement of the ewe in the pen. As injection of steroid has been shown to have effects on lung function within 24–48 h (Lanteri et al. 1994), we chose to a 48 h infusion to test our hypotheses.

Before the start of the infusion, and after 48 h of infusion, fetal and maternal blood samples (7 mL) were withdrawn to measure arterial PO_2_, PCO_2_, and pH (ABL77; Radiometer America, Westlake, OH), as well as plasma cortisol (Cortisol EIA, enzyme‐based immunoassay; EA65, Oxford Biomedical, Oxford, MI and aldosterone concentrations (aldosterone coat‐a count radioimmunoassay; RIA; TKAL2, Siemens, Deerfield, IL). Samples for cortisol assay were extracted in ethanol prior to assay. The cortisol assay has a minimal detectable concentration of 0.4 ng mL^−1^, and has minimal cross‐reactivity with betamethasone, progesterone or aldosterone (≤0.05%); the aldosterone assay has no cross‐reactivity with cortisol or the synthetic glucocorticoids. The aldosterone concentrations were analyzed in two RIAs with a coefficient of variation between assays of 15%; the cortisol analyses were performed in three plates with a coefficient of variation among plates of 16%.

Effects of steroid infusions on plasma hormones were analyzed for effects of time, aldosterone, and betamethasone treatment by three‐way analysis of variance (ANOVA). Differences among treatment groups were compared with Duncan's test. There were no effects of infusion of steroids on steroid hormone concentrations in the uninfused twins.

### In situ lung compliance

At the end of the infusions, the ewe and fetus(es) were killed (Euthasol, Virbac AH, Fort Worth, TX). In situ lung compliance was assessed by measurement of pressure changes during four stepwise injections of 10 mL of air into the trachea. The fetal chest was opened and a cuffed 4 mm endotracheal tube was inserted into the trachea to a point just above the bifurcation of the trachea, and cuff was inflated. The bronchus to the upper lobe of the right lung was clamped, and the endotracheal tube was connected via a three‐way stop cock to both a 60 mL syringe and to a pressure transducer (Transpac; Hospira, Lake Forest, IL). Pressure measurements were recorded in real time using LabView software (National Instruments, Austin, TX). With the chest wall open, lung compliance was then determined by measuring airway pressure responses to injections of four 10 mL boluses of air into the endotracheal tube at 10 s intervals; pressure was recorded at the end of each bolus. At the end of the steps, intrapulmonary pressure was then equilibrated to room pressure for 30 s, after which a second series of inflations were performed.

Pulmonary airway pressure data were analyzed by determining peak pressures at each inflation volume and were analyzed by three‐way ANOVA corrected for repeated measures using SPSS software (SPSS, IBM Corp, Armonk, NY); differences in pressures at each step in volume were compared by Newman Keul's test. The relaxation curve following each volume of inflation was fit to a three‐parameter exponential decay regression curve: *y* = *y*0 + a*e^−bx^, and the value of the parameter *y*0, representing the steady‐state pressure in the relaxation phase after each inflation was determined. Differences among treatment group for both peak pressure and *y*0 were analyzed by three‐way ANOVA corrected for repeated measures. Differences in the peak and plateau pressures after the initial 10 mL bolus of air were also compared using two‐way ANOVA using Sigmaplot software (Systat Software, Inc., San Jose, CA). Compliance at each step increase in volume was calculated and analyzed by three‐way ANOVA using Sigmaplot.

Samples of lung from the uninflated lobe were collected, flash frozen, and stored at −80°C for subsequent mRNA and protein determinations. A sample from the right lobe of the lung both before and after inflation was collected for histology by immersion fixation in 4% buffered paraformaldehyde. Tissues were processed for paraffin embedding. The entire left lobe was collected for determination of wet and dry weights.

### Immunoblotting

Relative expression of the ENaC‐*α* and ENaC‐*β* were assessed in whole cell and membrane‐enriched lung homogenates by immunoblot (Jesse et al. 2009; Keller‐Wood et al. 2009) using specific antibodies against the *α*‐subunit (used at 1:100, 3464; AbCam, Cambridge, MA) and *β*‐subunit (used at 1:1000, ENaCb21‐A; Alpha Diagnostics, San Antonio, TX) of ENaC. The blots were analyzed with a Chemi‐Doc system and Quantity One software (Bio‐Rad, Hercules, CA). We have identified two bands with molecular weights consistent with the predicted mature and immature forms of ENaC‐*α* protein (68 and 100 kDa, respectively), and ENaC‐*β* protein (112 and 102 kDa, respectively) and specificity of the bands was confirmed in experiments in which the antibody was preabsorbed to the immunizing antigen (Jesse et al. 2009). All tissue homogenates were run on four gels that were transferred and developed simultaneously. Values were expressed as optical density relative to Ponceau S staining of total protein, and analyzed by two‐way ANOVA with post hoc comparisons by Duncan's test.

### Histology and immunohistochemistry

Fixed, paraffin‐embedded lung sections from the uninflated lobe of the lung were stained for collagen (picrosirius red) or elastin (Miller's solution). Images of 10 fields per collagen or elastin‐stained section were photographed, avoiding fields containing major blood vessels; the average percent stained area was calculated (Image J software; NIH, Bethesda, MD) and analyzed by two‐way ANOVA. Total percent area of tissue was determined after applying a threshold using green RGB stack images (Image J). The threshold excluded airspace, but did not distinguish between alveolar space and alveolar duct space.

Antibodies used in Immunofluorescence staining to localize MR and ENaC‐*α* proteins were anti‐ENaC*α* (1:1000; ab65710, Abcam) with Alexa Fluor 594 secondary and anti‐MR (against MR‐18, 6G1: 1:40; courtesy of Dr. Gomez‐Sanchez et al. 2006) with Alexa Fluor 488 secondary (1:500; Invitrogen, Carlsbad, CA). Nuclei were stained with Hoechst 33342 (Invitrogen). This MR antibody is directed to a highly conserved portion of MR which is identical in rat, mouse, and human and differs by one amino acid from the predicted ovine sequence. This antibody stains nuclear MR in rodent tissues (Gomez‐Sanchez et al. 2006) and in ovine hypothalamus and heart (Reini et al. 2008; Keller‐Wood et al. 2011); in hippocampus from adrenalectomized rats, the band at the predicted molecular weight of 107kD is detected in cytosol using immunoblot techniques (Crochemore et al. 2005).

### Quantitative RT‐PCR

RNA was purified from lung samples collected from each fetus using Trizol (Ambion, Carlsbad, CA), followed by further purification with RNeasy Plus kits (Qiagen, Valencia, CA). The RNA extracts were tested, and found negative, for the presence of genomic DNA contamination. Expression of *β*‐actin (ACTB), SGK), ENaC‐*α* (SCNN1A), ENaC‐*β* (SCNN1B), Na,K ATPase‐*α*1 (ATP1A1), aquaporins (AQP1 and AQP5) (Jesse et al. 2009), and surfactant proteins (SP‐A, SFTPA1; SP‐B, SFTPB; and SP‐C, SFTPC) were determined using Taqman chemistry with cDNA (High Capacity Reverse Transcriptase kits, ABI, Foster City, CA). Probes and primers for the surfactant proteins were designed from ovine sequences (Primer Design software; ABI, Foster City, CA) and are shown in Table 1; primers for the remaining genes have been previously published (Liu et al. 2003; Jesse et al. 2009; Keller‐Wood et al. 2009). Expression of each gene was analyzed by the ΔC_t_ method (Jesse et al. 2009) and were normalized against the beta actin signal in the same sample. These values were analyzed by two‐way ANOVA and group differences were compared by Student–Newman–Keuls method. Results were expressed as fold changes relative to control fetuses.

**Table 1.** Forward and reverse primers for genes used in real‐time PCR analysis

Official symbol	OMIM number	Forward primer	Reverse primer	Probe FAM‐TAMRA
AKR1C3	603966	GTGATTCGGTGGATCTCTGTCA	GCCCTGCATCCTTACACTTCTC	
CATHL1B or CAMP	443312 600474	TGCTGTGGATCAGCTCAATGA	CAGCTCGAGAAGACGGTAAATGT	
CXCR4	162643	TGGCGGACCTCCTCTTTG	CCAGTTTGCCACAGCATCAA	
GZMB	123910	TCGGGCCTTCACCAAAGTC	TCAGAGGCTTTTCATGGTTTTCTT	
NCAPH	602332	CACCTGCAACAACGCAAGAC	TGACTCCCTGTAAGTGGTGATGTC	
PRG3	606814	CTGGATCGGAGGTCAGTTACG	TAAAATTCCAACAACTCCCATCAG	
SC5	443424	CCGCGGAGCAGTGTGACT	TGACTGTCCCCACACACTCTTT	
SFTPA1	178630	TGACCCTTATGCTCCTCTGGAT	CAGGGCTTCCAAGACAAACTTC	TTCTGGCCTCGAGTGCGACACAAA
SFTPB	178640	TCCCTGCCTGGAGAATGG	CTGCCTGAGTGGTCACAAACA	TGCCACAAGTCTCTGAGTGCCAGCTCT
SFTPC	178620	GAACCTGCTGCTACATTATGAAGGT	GAAGTTCGGCAATTTTCTAGTGAGA	TCCGCAGAGCATCCCAAGTCTCGA

### Genomic array

Four sets of twin fetuses at 130‐days gestation were used; in each set one of the pairs was treated with Aldo (0.2 mg over 48 h) and the twin was untreated. RNA was extracted from frozen lung tissue of the uninflated lobe as described above, purified, labeled with Agilent one color Quick Amp labeling kits and hybridized to Agilent ovine 8X15k gene expression microarrays (Agilent 02519921; Wilmington, DE). The array data were analyzed by ANOVA using JMP Genomics software (SAS Institute, Cary, NC [Cline et al. 2007]) and pathway analysis was performed with Cytoscape using the Genemania (Warde‐Farley et al. 2010), ClusterOne (Bader and Hogue 2003), and BiNGO (Maere et al. 2005) plugins as described previously (Rabaglino et al. 2012). The data discussed in this publication have been deposited in NCBI's Gene Expression Omnibus (Edgar et al. 2002) and are accessible through GEO Series accession number GSE53048 (http://www.ncbi.nlm.nih.gov/geo/query/acc.cgi?acc=GSE53048).

Real‐time PCR analysis was used to confirm the microarray data for selected genes, including CATHL1B, SC5, NCAPH, CXCR4, GZMB, and AKR1C3, using Sybr green chemistry. Primer sets for CATHL1B (named CAMP in Homo sapiens), SC5 (unique to Ovis aries), AKR1C3, NCAPH, GZMB, PRG3, and CXCR4 were designed from ovine sequences (Primer Design software; ABI; Table 1). Template cDNA concentration and reaction efficiency were validated for newly designed primer or primer probe sets using pooled lung cDNA from control animals. Differences in gene expression between control and aldosterone‐infused twins were compared by paired *t*‐test. A significance level of *P* < 0.05 was accepted throughout the analyses. Expression of SC5, NCAPH, CXCR4, GZMB, and AKR1C3 1 and 11bHSD2 were also analyzed in the larger set of animals making up all 6 groups studied (control: *n* = 11, aldosterone: *n* = 5, 0.25Beta: *n* = 4, 0.75Beta: *n* = 4, Aldo/0.25Beta: *n* = 4, Aldo/0.75Beta: *n* = 4) and the expression of each gene was analyzed by the ΔC_t_ method (Jesse et al. 2009) and were normalized against beta actin in the same sample. These values were analyzed by two‐way ANOVA and group differences were compared by Student–Newman–Keuls method.

## Results

### In vivo measurements

Plasma aldosterone was increased in all fetuses infused with aldosterone (Table 2). Betamethasone infusion significantly reduced plasma aldosterone concentrations in the betamethasone‐treated fetus as compared to control fetuses. There were no differences in fetal plasma cortisol concentrations among groups (Table 2).

**Table 2.** Cortisol and Aldosterone concentrations at the start and end of control or aldosterone (A) and/or betamethasone (Beta) infusion in ovine fetuses

Treatment group	Cortisol (ng mL^−1^)		Aldosterone (pg mL^−1^)	
	0 h	48 h	0 h	48 h
Control	5.2 ± 1.0	6.3 ± 2.0	179 ± 50	236 ± 38
A	7.1 ± 2.0	6.0 ± 2.0	177 ± 35	997 ± 90^1^^,^^2^
0.25Beta	2.5 ± 0.9	2.5 ± 1.2	122 ± 63	110 ± 31^2^
A/0.25Beta	3.5 ± 0.5	2.6 ± 0.8	161 ± 72	739 ± 204^1^^,^^2^
0.75Beta	5.1 ± 1.6	5.9 ± 0.9	108 ± 28	110 ± 21^2^
A/0.75Beta	3.6 ± 1.5	4.4 ± 1.3	86 ± 31	861 ± 151^1^^,^^2^

Data are expressed as mean ± SEM.

Doses of betamethasone (0.25 or 0.75 mg) are indicated as 0.25Beta and 0.75Beta, respectively.

^1^ Indicates significantly different than 0 h in same group of fetuses.

^2^ Indicates significantly different than control group, *P* < 0.05.

### Fetal lung inflation pressures

**Figure 1.**
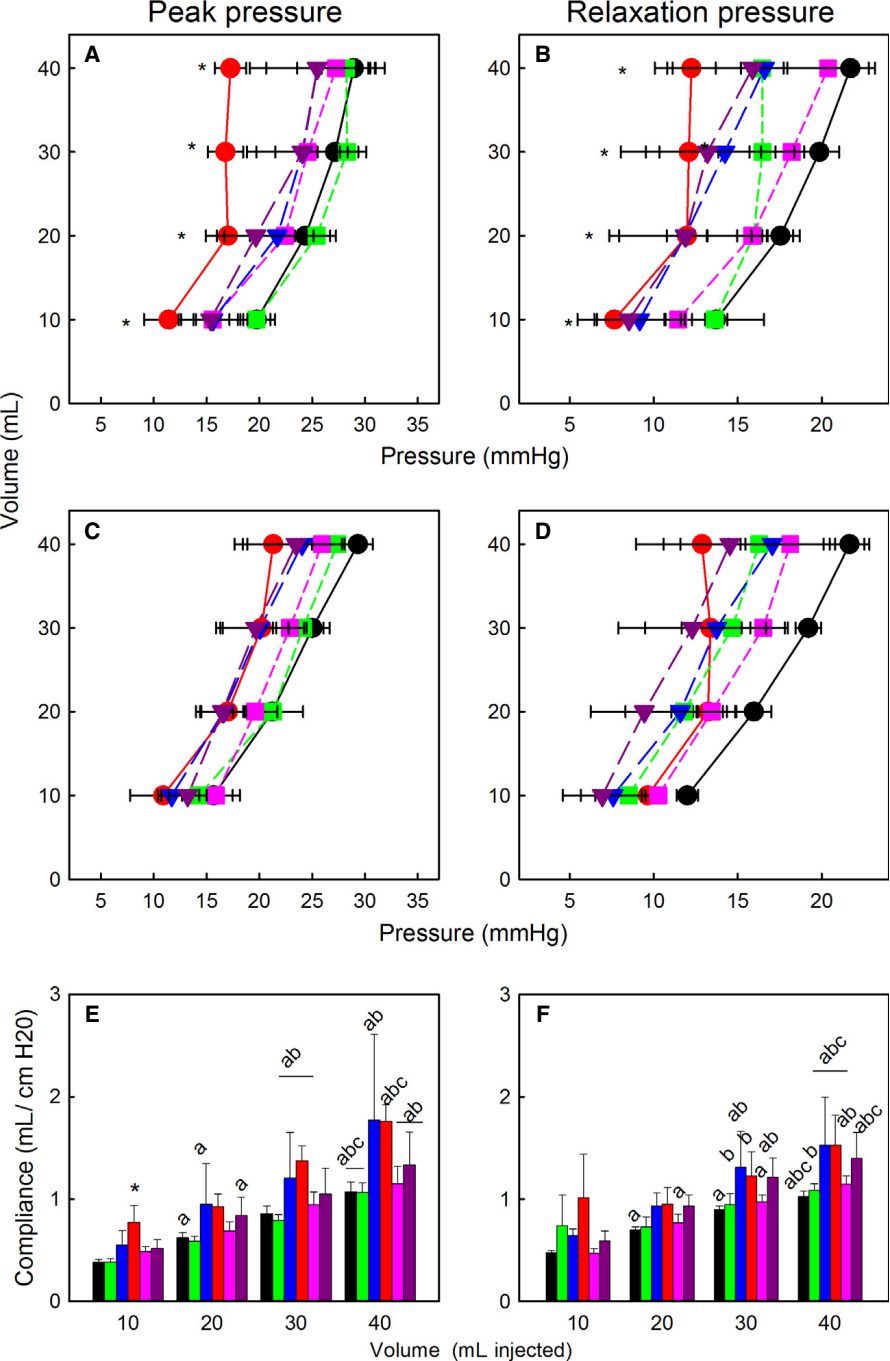
(A–D) Volume–Pressure relationships in ovine fetal lung after treatment with Aldo (red circles and bars, *n* = 5), 0.25Beta (green squares and bars, *n* = 4), 0.75Beta (blue triangles and bars, *n* = 4), and Aldo/0.25Beta (pink squares and bars, *n* = 4), Aldo/0.75Beta (purple triangles and bars, *n* = 4), or control twins (black circles, *n* = 9). Panels A and C, peak pressure changes during first and second sets of inflations, respectively; Panels B and D, steady‐state relaxation pressures during first and second sets of inflations, respectively. *indicates pressure in aldosterone‐treated lungs different than control group at same volume, *P* < 0.05 Panels E–F, calculated compliance during inflation using peak pressure and injected volumes during the first (E) and second (F) sets of inflations. a, different than 10 mL; b, different from 20 mL; c, different from 30 mL during same series of inflations. *indicates different from control group at same volume. All data are expressed as mean ± SEM.

As expected there were significant overall effects of volume on peak pressure and on plateau relaxation pressures during serial lung inflations. Contrary to our hypothesis, there was no overall effect of aldosterone either on peak inflation pressures, plateau relaxation pressures or on calculated lung compliance (Fig. 1A–E). However, the initial peak pressure after injection of 10 mL of air (11.4 ± 2.3 vs. 19.8 ± 1.3 mmHg in controls) and the compliance at this volume were significantly reduced in fetuses after infusion of aldosterone (Fig. 1A and E). The relatively low doses of betamethasone used in this study did not alter inflation or relaxation pressures, and contrary to our hypothesis it did not amplify the response to aldosterone. In control and betamethasone‐infused fetuses, sequential steps in volume produced stepwise changes in peak pressure from 10 to 30 mL, whereas aldosterone did not significantly increase peak pressures as volume increased from 20 to 40 mL (Fig. 1A). During the second inflation series, all fetuses except those treated with aldosterone alone showed significant step increases in peak pressure with increases in volume from 10 to 40 mL; after infusion of aldosterone, a plateau in peak pressure was achieved by 20 mL (Fig. 1C).

Similarly, in both the first and second inflation series, there was a progressive increase in the plateau relaxation pressures after each step increase in volume in the control fetuses, but after aldosterone treatment, the relaxation pressures after 20**–**40 mL were not significantly different (Fig. 1 B and D). During the first inflation series, 0.25Beta pressures at 10**–**20 and 30**–**40 mL were not different; 0.75Beta produced progressive increases in pressure. During the second series of inflations, in control and 0.25Beta fetuses the increases were progressive from 10 to 40 mL, whereas after 0.75Beta or Beta/Aldo, there were increases from 10 to 30 mL.

### Gene and protein expression

**Figure 2.**
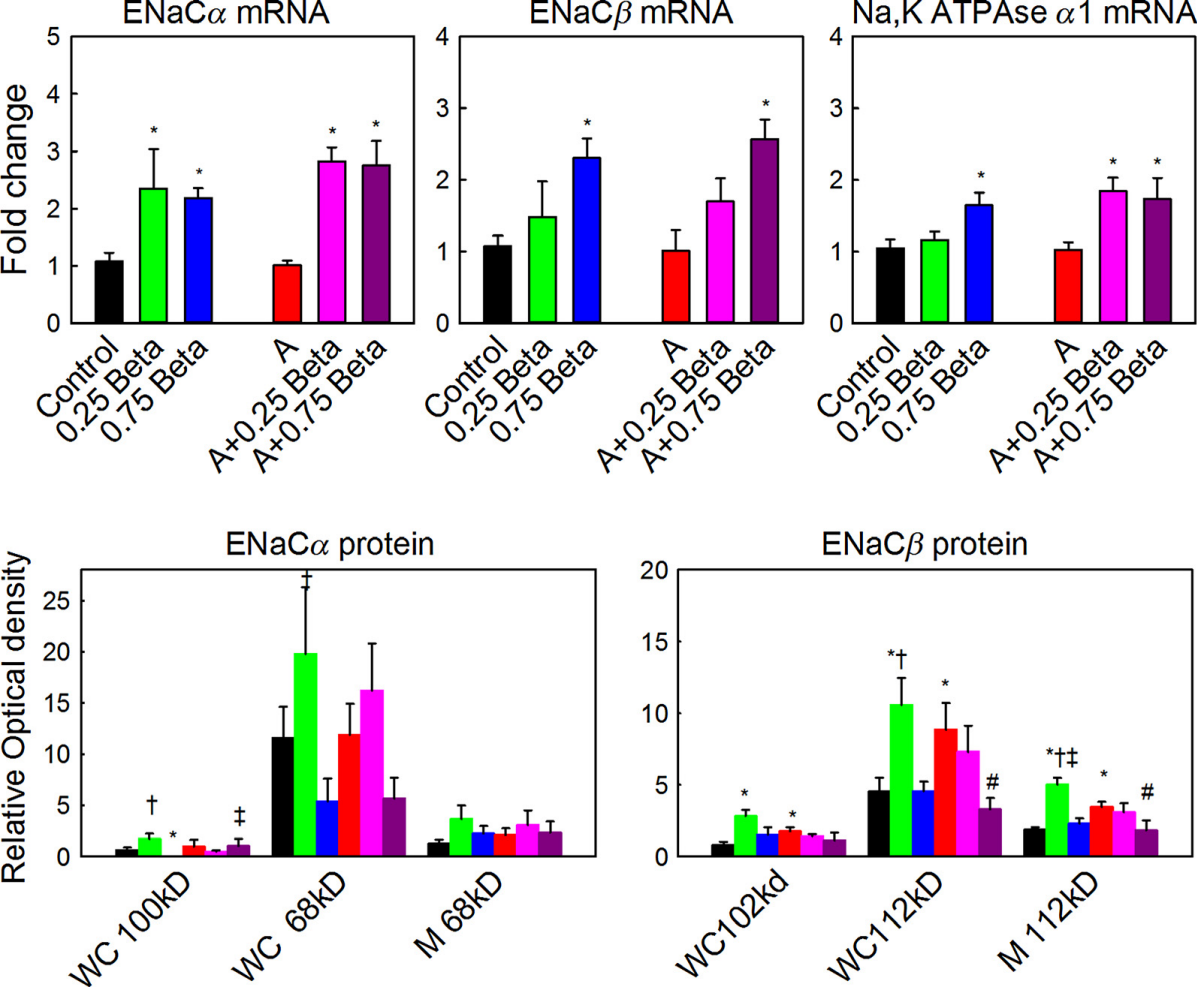
Top panels: Relative fold changes in mRNA of ENaC‐*α*, ENaC‐*β*, and Na,K ATPase‐*α*1. Expression of mRNA is expressed as mean fold change in each group (indicated below each bar) relative to expression in the control group: black bars, betamethasone (Beta); green bars, 0.25Beta; blue bars, 0.75Beta; red bars, aldosterone (Aldo), pink bars, Aldo/0.25Beta; purple bars, Aldo/0.75Beta. Lower panels: Changes in ENaC‐*α* and ENaC‐*β* protein expression. Protein was quantified as optical density of bands with equal loading of membrane (M) and whole cell (WC) protein (70 μg); estimated molecular weights for each band that was quantified are indicated. Differences between groups for each measure are indicated: *different from control; ^#^different from aldosterone alone; ^†^differences between 0.25Beta and 0.75Beta treatment with the same aldosterone treatment; ^‡^difference between with aldosterone and without aldosterone treatment at same dose of betamethasone; *P* < 0.05. n's are as in Fig. 1.

The results do not support a role of aldosterone in regulation of genes known to be involved in the reabsorption of lung fluid. As expected, betamethasone significantly increased expression of ENaC‐*α*, ENaC‐*β*, and Na,K ATPase‐*α*1 mRNA in lung (Fig. 2). Aldosterone infusion did not alter the expression of these genes. However, both doses of betamethasone increased Na,K ATPase*α*1 when coinfused with aldosterone, only 0.75Beta increased the expression in the absence of increased aldosterone. Expression of SGK mRNA did not differ in any group relative to the control group.

**Figure 3.**
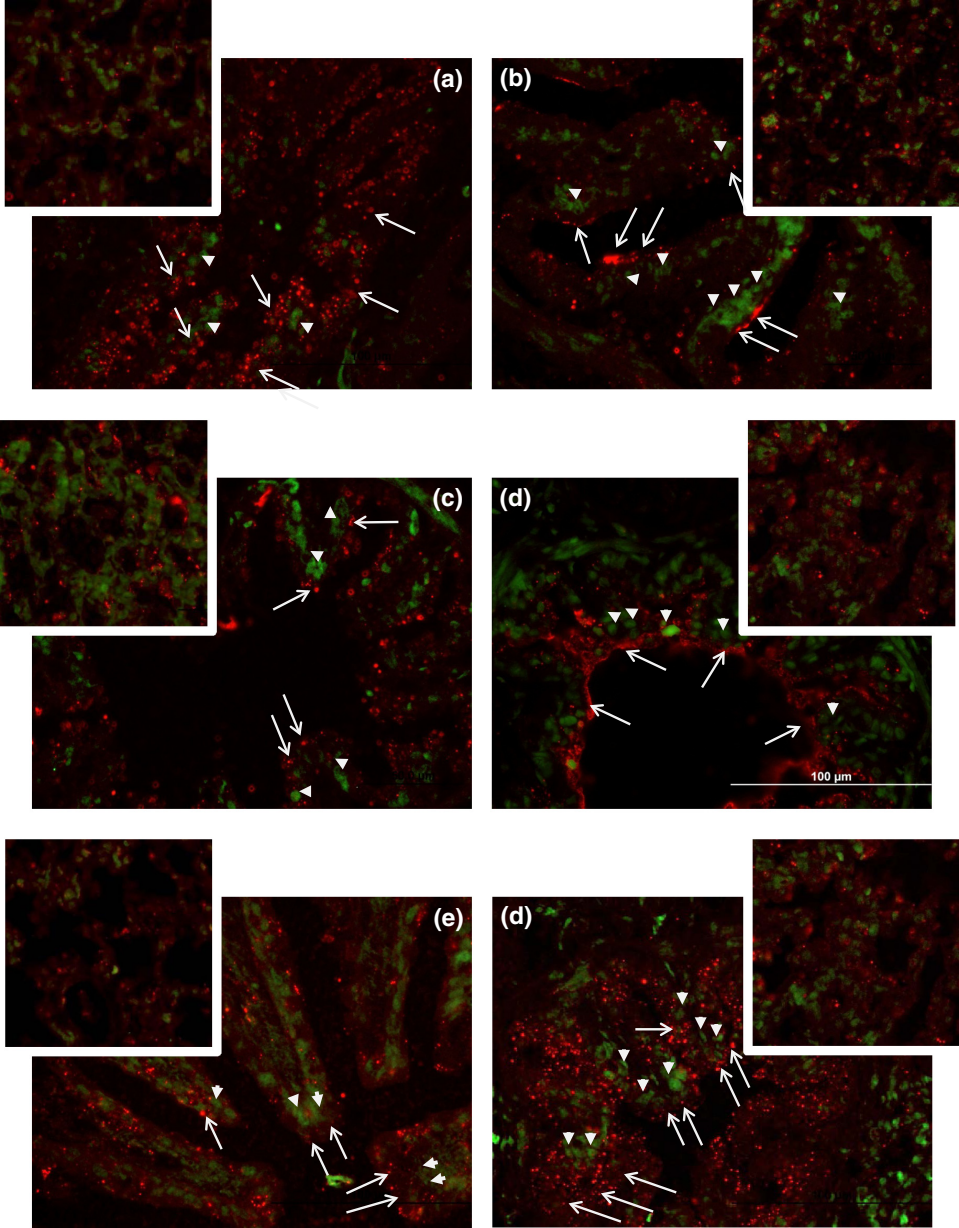
Representative images of lung depicting MR (green) and ENaC*α* (red) localization in lungs from (A) control fetuses, or fetuses after infusion of: (B) 0.25Beta, (C) 0.75Beta, (D) Aldo, (E) Aldo/0.25Beta, and (F) Aldo/0.75Beta. Large airway staining is shown in the large image in each panel; the smaller inserts are images of alveoli. All images are shown at 400×; the scale for all figures is shown by the white bar in panel E. Examples of MR staining are shown with solid arrowheads and ENaC‐*α* is shown with open arrowheads with long tails.

Neither betamethasone nor aldosterone in the doses used increased expression of ENaC*α* subunit protein in the whole cell homogenates from the lungs. In lung whole cell homogenates, there was a significant effect of betamethasone on the abundance of the 68 and 100 kD forms of ENaC‐*α* protein (Fig. 2), however this effect was to decrease protein after 0.75Beta compared to 0.25Beta. There was no effect of aldosterone on expression of either mature or immature forms of the ENaC‐*α* protein. The expression of mature ENaC‐*α* in membrane‐enriched fractions was not changed by any of the steroid treatments; there was no detectable immature ENaC‐*α* protein in membrane‐enriched samples. Because aldosterone did not alter the gene or protein expression of ENaC‐*α*, we verified the presence of MR in ENaC‐*α*‐expressing cells; immunohistochemistry revealed that ENaC‐*α* was found in many of the cell‐expressing MR, including both epithelial cells of the large airways and alveolar epithelium (Fig. 3).

Infusion of aldosterone increased the level of mature ENaC‐*β* protein in both whole cell and membrane preparations, and the immature ENaC‐*β* in whole cell extracts as compared to control fetuses. There were significant effects of betamethasone alone and significant aldosterone–betamethasone interactions on expression of the 112 kD form of ENaC‐*β* protein in membrane‐enriched or whole cell extracts. There was also a significant betamethasone–aldosterone interaction on the immature (102 kD) ENaC‐*β* protein in whole cell homogenates (Fig. 2). The expression of both forms of ENaC‐*β* protein in whole cell, and of the mature form in membrane‐enriched extracts, was increased in lungs of fetuses after 0.25Beta compared to control, however 0.75Beta fetuses did not increase the ENaC‐*β* in whole cells, and infusion of aldosterone with 0.25Beta did not increase the expression of ENaC‐*β* protein.

There were no overall effects of these doses of betamethasone or aldosterone on the expression of MR, GR, AQP‐1, AQP‐5, SP‐A, SP‐B, or SP‐C mRNAs in the fetal lung at 130 days (Table 3). The higher dose of betamethasone produced a similar increase in expression of both 11*β*HSD1 and 11*β*HSD2 in fetal lung.

**Table 3.** Expression of mRNA for glucocorticoid receptor (GR), mineralocorticoid receptor (MR), aquaporins 1 and 5 (AQP1, AQP5) in lungs after infusions aldosterone (A), betamethasone (Beta) or aldosterone and betamethasone (A+Beta)

	GR	MR	AQP1	AQP5	SGK1	SP‐A	SP‐B	SP‐C	11*β*HSD1	11*β*HSD2
Control	1.02 ± 0.07	1.02 ± 0.08	1.08 ± 0.16	1.03 ± 0.09	1.02 ± 0.07	1.12 ± 0.21	1.05 ± 0.12	1.08 ± 0.20	1.05 ± 0.11	1.10 ± 0.13
A	0.87 ± 0.13	0.85 ± 0.11	0.99 ± 0.13	1.04 ± 0.17	1.07 ± 0.13	1.96 ± 0.91	1.42 ± 0.42	1.10 ± 0.25	1.06 ± 0.24	1.47 ± 0.21
0.25Beta	0.71 ± 0.17	0.64 ± 0.18^1^	1.13 ± 0.21	1.37 ± 0.43	1.18 ± 0.09	2.00 ± 1.06	1.10 ± 0.31	0.63 ± 0.19*^,^^2^^,^^3^	1.32 ± 0.30	1.61 ± 0.28
A/0.25Beta	1.10 ± 0.08	0.75 ± 0.06	1.24 ± 0.25	1.25 ± 0.07	1.22 ± 0.18	1.71 ± 0.42	1.59 ± 0.19	1.34 ± 0.07	1.92 ± 0.33	1.82 ± 0.19
0.75Beta	0.73 ± 0.07	0.76 ± 0.10	1.51 ± 0.20	1.26 ± 0.09	1.02 ± 0.16	1.17 ± 0.15	1.17 ± 0.11	1.63 ± 0.41	2.15 ± 0.31^1^	1.94 ± 0.20^1^
A/0.75Beta	0.70 ± 0.14	0.81 ± 0.08	1.01 ± 0.07	1.17 ± 0.15	1.01 ± 0.17	1.60 ± 0.28	1.35 ± 0.36	1.25 ± 0.13	1.63 ± 0.43	1.90 ± 0.58

Data are expressed as mean fold change ± SEM relative to the control group mean.

^1^ Indicates significantly different than control group.

^2^ 0.25Beta versus 0.75Beta, same aldosterone treatment.

^3^ With aldosterone versus without aldosterone treatment at same dose of betamethasone; *P* < 0.05.

### Lung histology

**Figure 4.**
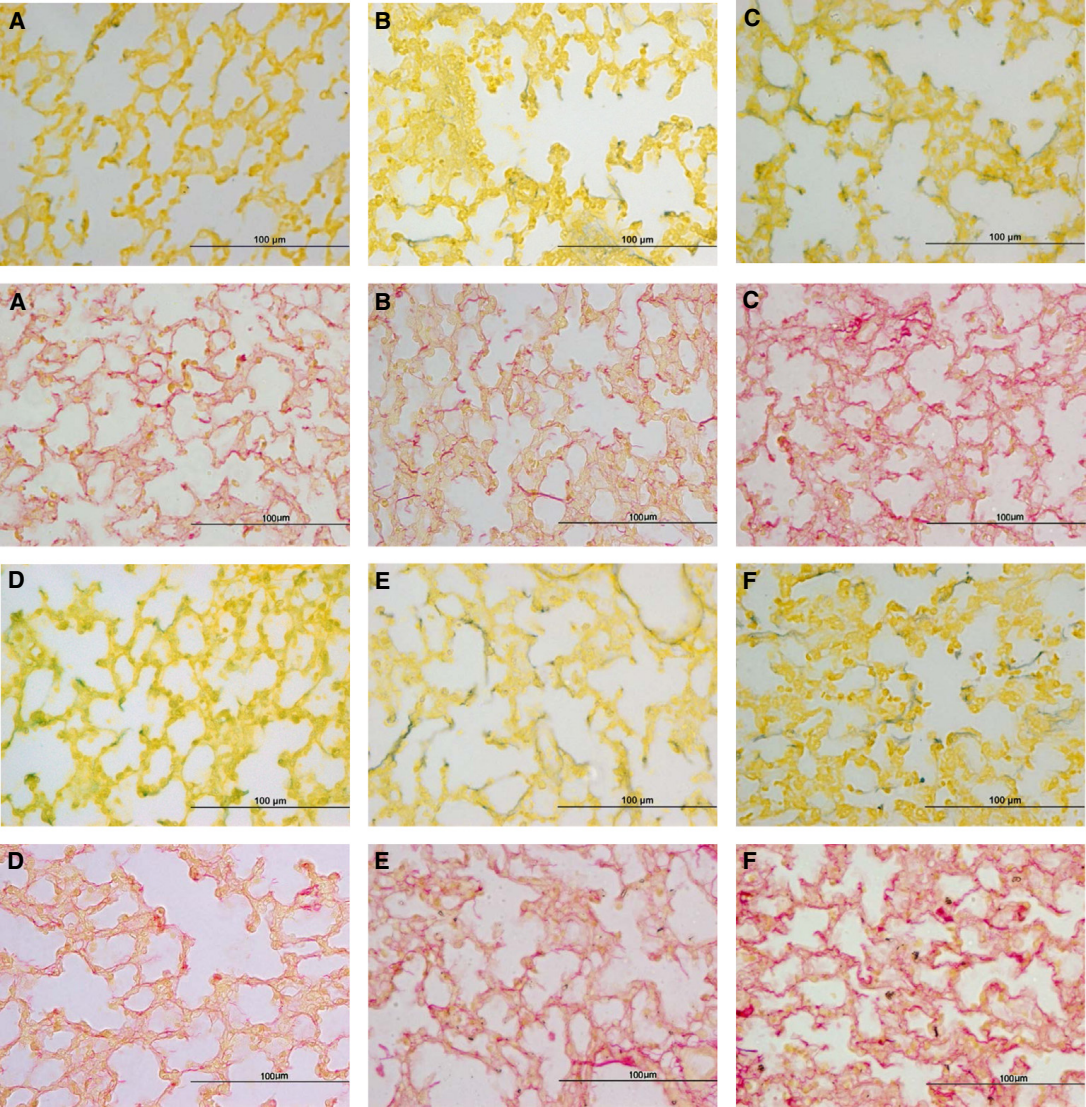
Representative images of staining for elastin (black against yellow tissue staining; upper panels within each set) and collagen (red, in lower panels in each set) in lungs from (A) control fetuses, or fetuses after infusion of: (B) 0.25 mg Beta, (C) 0.75 mg Beta, (D) Aldo, (E) Aldo/0.25Beta, and (F) Aldo/0.25Beta.

There was no significant change in the wet weight or relative wet to dry weight ratio of the left lobe of the lung between corticosteroid infused and control fetuses, nor was left lobe weight relative to body weight different among the groups. Betamethasone, but not aldosterone, significantly increased elastin and collagen staining in lung sections (Fig. 4, Table 4). Collagen and elastin in lung from 0.75Beta fetuses were significantly greater than that in control (Table 4). Elastin was also increased after Aldo/0.75Beta (Table 4).

**Table 4.** Elastin and collagen density in lungs before and during infusions aldosterone (A), betamethasone (Beta) or aldosterone and betamethasone (A+Beta)

	Elastin content (% area)	Collagen content (% area)
Control	1.23 ± 0.16	7.70 ± 0.91
A	1.43 ± 0.09	6.92 ± 2.21
0.25Beta	1.56 ± 0.32	6.36 ± 0.32
A/0.25Beta	1.90 ± 0.19	7.41 ± 0.90
0.75Beta	2.16 ± 0.19^1^	11.37 ± 1.36^1^,^2^
A/0.75Beta	2.64 ± 0.24^2^,^3^	10.68 ± 1.32

Data are expressed as mean ± SEM.

^1^ Indicates significantly different than control group.

^2^ 0.25Beta versus 0.75Beta, same aldosterone treatment.

^3^ Versus aldosterone alone.

### Genomic array results

**Figure 5.**
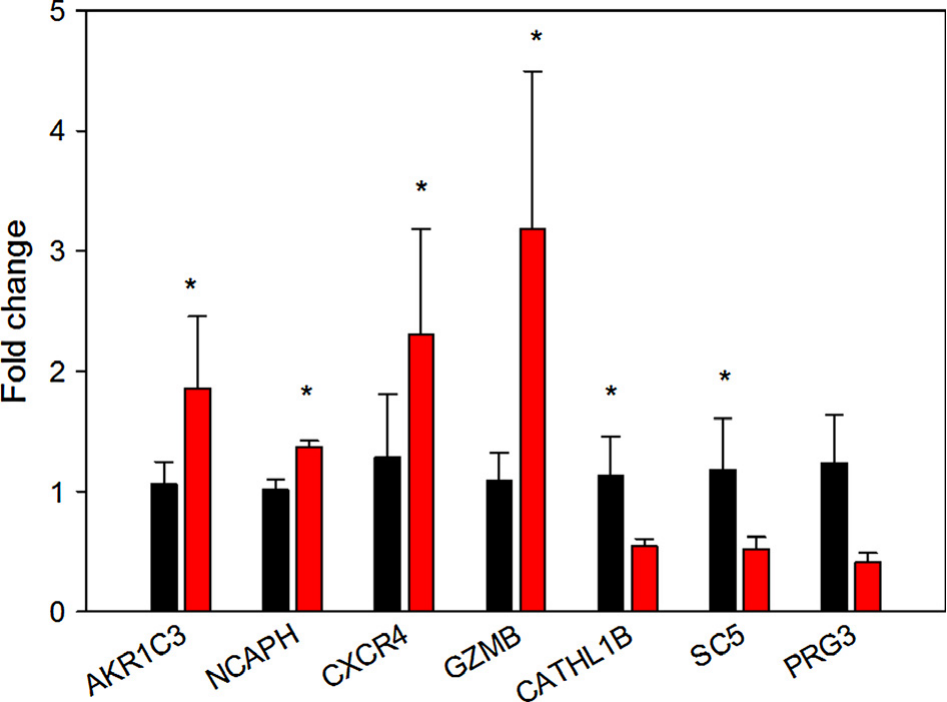
Fold changes in gene expression in four sets of twin pregnancies with one aldosterone‐treated fetus (red bars) relative to the mean values in the control twins (black). *difference between groups, *P* < 0.05.

Of 688 probes with unique and known gene names which were significantly differently expressed between the control and Aldo twins at *P* ≤ 0.05, 220 genes were upregulated and 468 genes were downregulated. There were a large number of genes in both the up‐ and downregulated pathways involved in the cell cycle (Table 5). Upregulated genes included 42 cell cycle genes, 16 cell division‐related genes, and 32 DNA metabolic process‐related genes; downregulated genes included 108 genes associated with negative regulation of cell processes and 47 genes related to cell cycle‐related processes. Real‐time PCR confirmed the significant increase in expression of NCAPH, which encodes a protein associated with condensed DNA, confirming the effect on cycle (Fig. 5). Real‐time PCR confirmed that aldosterone differentially expressed several genes that were identified in the array analysis and for which other investigators have described roles in lung maturation and/or innate immunity (Table 6). These included a decrease in expression of genes associated with lung stiffness (CATHL1B in sheep; similar to CAMP in humans and SC5), innate immunity, and response to injury (CXCR4 and GZMB), and steroid hormone and prostaglandin metabolism (AKR1C3). There was a tendency for the expression of PRG3 – a gene identified on the array and associated with immune cell function – to be increased, however as this was only increased in three of the four aldosterone‐treated twins, this result was not statistically significant (*P* = 0.125).

**Table 5.** Aldosterone‐regulated biological processes or molecular functions in the lung

	Number of genes	*P*
Upregulated		
ATP binding	34	1.59×10^−2^
DNA repair genes	16	6.4×10^−3^
Mitosis	15	6.4×10^−3^
Nuclear division	15	6.4×10^−3^
Cell cycle checkpoint	12	6.4×10^−3^
S phase of mitotic cell cycle	11	4.0×10^−4^
G1/S transition of mitotic cell cycle	10	2.03×10^−2^
G2/M transition of mitotic cell cycle	8	2.57×10^−2^
M/G1 transition of mitotic cell cycle	8	1.1×10^−3^
Helicase activity	7	3.26×10^−2^
DNA replication initiation	6	3.0×10^−4^
Histone‐lysine methyltransferase activity	4	2.18×10^−2^
Positive regulation of cell cycle cytokinesis	3	2.03×10^−2^
DNA replication‐dependent nucleosome assembly	2	9.9×10^−3^
Hexokinase activity	2	2.20×10^−2^
AMP response element binding	2	2.69×10^−2^
Downregulated		
Phosphate containing compound metabolic process	94	1.94×10^−2^
Macromolecular complex assembly	45	3.29×10^−2^
Cytoskeleton organization	39	2.48×10^−2^
RNA binding	39	2.31×10^−2^
Negative regulator of apoptotic process	33	1.24×10^−2^
Protein kinase binding	24	3.30×10^−3^
Ubiquitin‐dependent protein catabolic process	22	4.25×10^−2^
Protein serine/threonine phosphate activity	7	1.05×10^−2^
Negative regulator of TOR signaling cascade	5	1.24×10^−2^
RNA helicase activity	5	2.35×10^−2^
Microtubule plus‐end binding	4	6.20×10^−3^
Fatty acid homeostasis	4	1.24×10^−2^
Positive regulator of cholesterol biosynthetic process	3	1.94×10^−2^
Positive regulator of chromatin silencing	2	4.12×10^−2^
AcetylCoA carboxylase kinase	2	2.35×10^−2^
Hydroxymethylglutaryl‐CoA reductase (NADPH) kinase activity	2	2.35×10^−2^

Gene names that were statistically significant within each biological process can be found in Appendix.

**Table 6.** Genes with differential expression after aldosterone infusion into one twin, validated by qRT‐PCR

*GENE* Protein	Function	Up/down regulated
*AKR1C3*: 3a HSD type IIb (HSD17B5)	Androgen synthesis and metabolism; prostaglandin F synthase (Lukacik et al. 2006) localized in conducting epithelia and associated with human fetal lung maturation (Provost and Tremblay 2007)	Up
*CXCR4*: chemokine CXC motif receptor 4	Stem cell/progenitor cell marker (Kucia et al. 2004) Expressed on circulating and amniotic fluid stem cells (Carraro et al. 2008) Mediates response to epithelial injury in lung (Gomperts et al. 2006)	Up
*GZMB*: granzyme B	Susanto et al. (2012) role in apoptosis and cytokine production in cytotoxic T cells and NK cells; role in Regulatory T‐cell response to inflammation and infection (Loebbermann et al. 2012; Hirota et al. 2013)	Up
*NCAPH*: non‐SMC condensin I complex, subunit H	Required for chromosome condensation and chromatid segregation during mitosis (Cabello et al. 2001)	Up
*CAMP* (*ovine CATHL1B*): Cathelicidin antimicrobial peptide or LL37	Antimicrobial peptide (Tomasinsig and Zanetti 2005); cell chemotaxis and immune mediation increase epithelial cell stiffness; decrease epithelial permeability in lung (Byfield et al. 2011a,b)	Down
(*ovine SC5*): cathelin‐related prepropeptide		Down

Gene and protein names in this table are those for the homologous human gene or protein; unless indicated these are the same in ovis aries or bos taurus. Functions are as determined in the cited literature.

**Figure 6.**
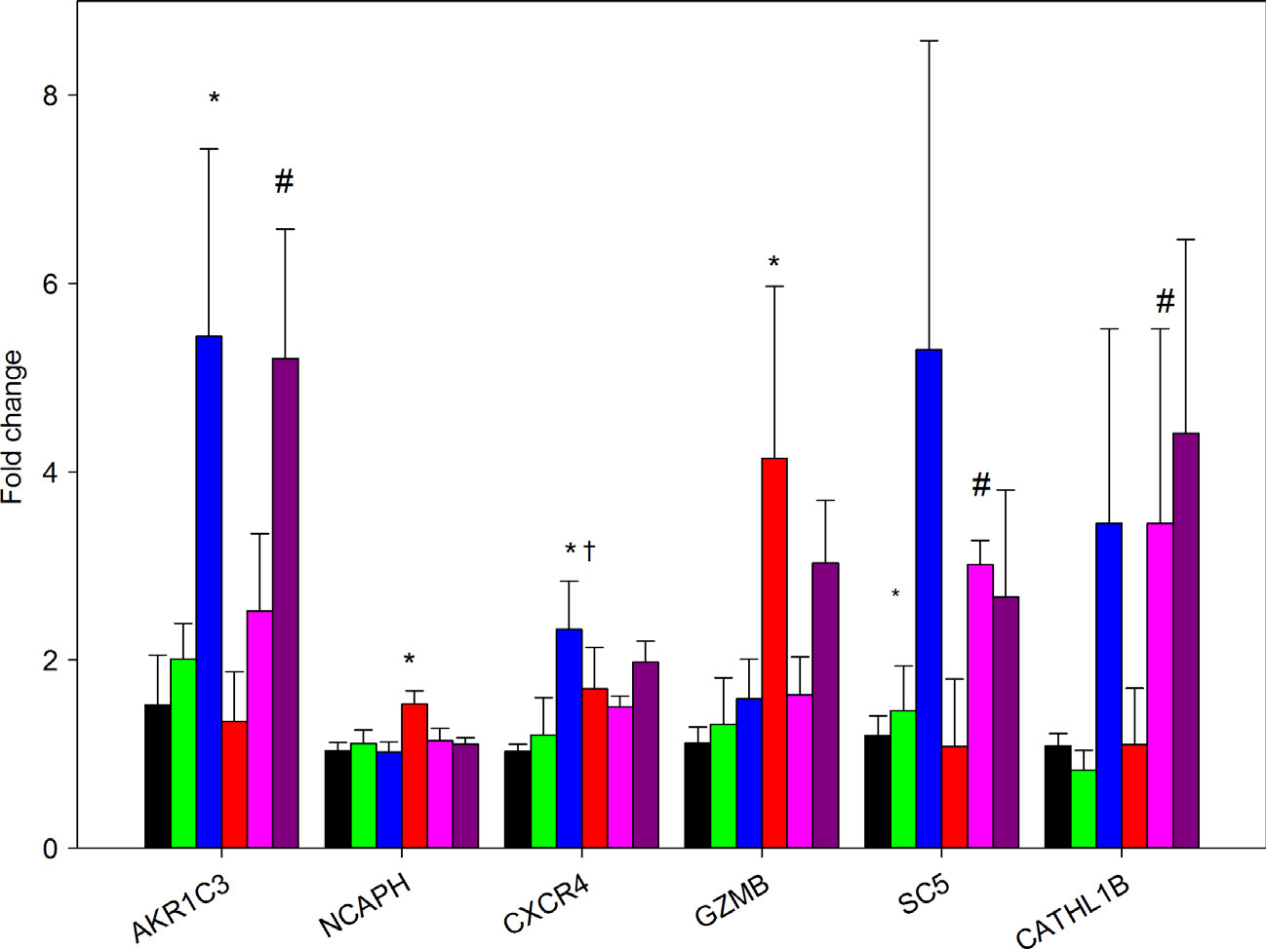
Fold changes in expression of AKR1C3, NCAPH, CXCR4, GMZB, SC5, and CATHL1B in the six groups of fetuses. Expression of mRNA is expressed as mean fold change in each group (indicated below each bar) relative to expession in the control group: black bars, betamethasone (Beta); green bars, 0.25Beta; blue bars, 0.75Beta; red bars, aldosterone (Aldo), pink bars, Aldo/0.25Beta; purple bars, Aldo/0.75Beta. *different from control; ^#^different from aldosterone alone; ^†^differences between 0.25Beta and 0.75Beta treatment with the same aldosterone treatment.

When these genes were compared across all six groups studied, an increased expression of AKR1C3 and CXCR4 were found in the lung from fetuses treated with 0.75Beta. Significant increases in both GMZB and NCAPH were found by two‐way ANOVA only in the lungs of the aldosterone‐treated fetuses (Fig. 6). There was no interaction between aldosterone and betamethasone treatment in any of the genes analyzed.

## Discussion

The results of this study indicate that MR plays a role in fetal lung function that is distinct from that of GR, but disprove the hypotheses that aldosterone would alter expression of genes important for sodium conductance across lung epithelium, and the hypothesis that aldosterone would synergize with submaximal doses of betamethasone in either gene expression or lung compliance. The effect of aldosterone occurs by a different mechanism than the effects of glucocorticoids, and does not appear to involve the induction of the sodium channels that are critical for lung liquid reabsorption. In this regard, the effects of the mineralocorticoid agonist are distinct from the well‐characterized transcriptional effects of MR on SGK, ENaC*α,* and Na,K ATPase in the kidney (Chen et al. 1999; Pearce et al. 2000). Instead, the actions of MR appear to be related to changes in cell cycle activity and may be associated with maturation of lung structure. Our results indicate an initial increase in ovine fetal lung compliance as a consequence of aldosterone treatment, which is apparent with the initial inflation of the lungs, and a greater initial stress relaxation. Our results predict that these pressure‐lowering effects of aldosterone are unlikely to be observed in lungs that had been ventilated, because the effect was limited to the first lung inflation. Thus, it appears that MR may play a role in preparing the lung for the first breath of life rather than to alter the ability to fully inflate the lung, which depends on both reabsorption of lung fluid and on surfactant production.

Glucocorticoid receptor agonists are known to increase surfactant production (Ueda et al. 1995), however our dose and time course were not sufficient to increase expression of the surfactants. This relatively low dose of betamethasone increased expression of ENaC‐*α*, ENaC‐*β*, and Na,K ATPase‐*α*1 in the lung. This result was not surprising as GR agonists are also known to induce expression of ENaC‐*α*, ENaC‐*β*, and Na,K ATPase mRNAs (Venkatesh and Katzberg 1997; Itani et al. 2002; Nakamura et al. 2002). With these doses of betamethasone, there was no overall effect to increase ENaC subunit protein expression; however in vitro studies have suggested that induction of protein may require increases in oxygen tension, as would normally occur with birth (Jain et al. 2001). Betamethasone also did not significantly induce surfactant protein genes, nor was lung compliance increased. Treatment with a bolus of betamethasone to the mother, as is used clinically, increases lung compliance measured in the preterm lung after 30 min of ventilation (Jobe et al. 2007), and this effect is also observed in animal models, including the sheep, in which a similar dose is administered (Ikegami et al. 1997). However, the GR agonist dose in our treatment paradigm is lower, and the initial concentrations of steroid achieved in maternal and fetal blood would be substantially lower than the one achieved after administration of the clinically used dose of betamethasone. Our study was designed to approximate the activation of GR which would occur with a more modest increase in corticosteroids, rather than the increase occurring during the surge in cortisol at the time of delivery. MRs increase ENaC‐*α* transcription and localization in the kidney (Bhargava and Pearce 2004), so we had hypothesized that glucocorticoid and mineralocorticoid action in the fetal lung might work coordinately to increase both transcription and localization of ENaC subunits. Counter to our hypothesis, there was no synergy between MR and GR occupancy in the lung in terms of genomic effects, or any of the variables measured.

The mature form of ENaC‐*β* in the whole cell and in the membrane was increased by aldosterone. The mature ENaC‐*β* protein is thought to be required for activity of the channel (Hughey et al. 2004). In renal epithelium, there is a pool of available ENaC‐*β* and ENaC‐*γ* which allow for functional assembly of the sodium channel after acute stimulation of ENaC‐*α* (Hager et al. 2001; Loffing et al. 2001), suggesting that aldosterone may play a role in preparing the epithelium for the later effects of GR stimulation at term. It is also possible that MR in lung might alter ENaC activity after posttranslational modification of existing protein. In renal epithelial cells, aldosterone increases sodium channel activity through increasing 4,5‐bisphosphate (PIP2) and phosphatidylinositol 3,4,5‐trisphosphate (PIP3), increasing apical localization of channel subunits, and increasing the number of open channels (Ma et al. 2007). Elastase activity, which is stimulated by aldosterone (Sweet et al. 2008), also stimulates ENaC activity (Adebamiro et al. 2007). Consistent with reports of MR localization in alveolar type II cells in the adult lung (Suzuki et al. 2001), we found MR throughout the lung parenchyma and airways in cells expressing ENaC*α*, therefore, MR are positioned to be able to indirectly increase ENaC activity.

Glucocorticoid receptor also exerts effects not seen with the MR agonist to alter collagen/elastin remodeling of the lung. Changes in collagen and elastin fiber abundance and/or organization can result in profound changes in the biomechanical properties of the lung (Schellenberg et al. 1987; Tanaka and Ludwig 1999). The higher dose of betamethasone increased expression of both collagen and elastin in lung, consistent with the known actions of betamethasone on fetal lung (Beck et al. 1981), however aldosterone alone had no effect on collagen or elastin staining.

Because our initial results suggested an unknown action of aldosterone that was not related to the induction of sodium channels, we used an ovine gene expression microarray to identify genes that were differentially expressed in lungs of fetal lambs after aldosterone infusion. This revealed previously unidentified MR‐activated genes related to pathways important for proliferation, immune function, and epithelial cell stiffness or permeability. Betamethasone also appeared to influence genes in pathways related to immune function and to permeability or stiffness, although in the case of CATHL1B and SC5, the effect of betamethasone was in the opposite direction to that found on the array for aldosterone. Since most of the studies of these genes in the lung did not involve fetal lungs, the role of these genes in the final steps of lung maturation at term is unclear. The effects on lung compliance unique to aldosterone treatment appear to be potentially mediated by changes in cell proliferation. This was revealed by cluster and pathway inferences of the microarray data and confirmed by RT‐PCR for the expression of NCAPH, a member of this gene cluster. NCAPH protein has been shown to be expressed at a constant level in cells but its transcription is restricted to proliferating cells, being the highest during the G phase of the cell cycle (Cabello et al. 2001). Thus, aldosterone appears to stimulate cell proliferation in the late gestation fetal lung. The other gene uniquely upregulated by aldosterone in our studies, GZMB, may identify the cell type that was undergoing proliferation. In adult human lungs, the type II pneumocytes express GZMB, but bronchiolar epithelial cells do not. Therefore we speculate that the increase in lung compliance seen in aldosterone‐treated animals was due to increased numbers of type II pneumocytes. However, further experimentation would be needed to confirm this supposition as both alveolar macrophages and lymphoid aggregates also express GZMB (Vernooy et al. 2007). Other potential explanations for the effect of MR on initial compliance could be changes in the extracellular matrix induced by GZMB (Buzza et al. 2005), but, again, confirmation of this is beyond the scope of this study.

In the other studies that were performed in this laboratory, we have evidence that MR promotes growth in the late gestation fetal heart; MR are nonfibrotic and proproliferative (Reini et al. 2008; Feng et al. 2013). Effects of corticosteroids through MR to reduce apoptosis have been observed in the adult rat hippocampus (Crochemore et al. 2005). This raises the possibility that aldosterone could alter lung growth, proliferation of alveolar cells, inhibition of apoptosis, and/or thinning of the alveolar epithelium. The transcriptomic response to aldosterone supports a proliferative effect, with promitotic genes upregulated and antigrowth genes downregulated. However as antiapoptotic genes were downregulated, this suggests that aldosterone may play a role in remodeling the fetal lung. Normally in late gestation, alveolar space increases through septation of saccules and formation of new alveoli. Septation and increased alveolar number normally occur at a time of relatively low cortisol or corticosterone in sheep or rats; higher levels of corticosteroids or glucocorticoids inhibit septation, but can cause thinning of alveolar walls after septation (Massaro and Massaro 1996). Thus, the transcriptomic pattern may reflect effects of aldosterone on multiple cell types contributing to the overall increased ability to open airways with the initial inflation of the lung.

Our results suggest an effect of aldosterone in the maturing lung, consistent with the relatively high expression of MR in the preterm lung. In the normal near‐term fetus, these effects are likely exerted by cortisol. The results clearly indicate that this effect is not directly analogous to MR effects on ENaCs or Na,K ATPase expression in the postnatal kidney; GR mediates these effects, indicating that normally the high cortisol concentrations at birth are necessary to decrease alveolar fluid production and reduce surface tension. On the other hand, MR effects in the preterm lung may parallel those in nonepithelial tissues in which MR appear to affect cell proliferation: transcriptomic analysis also suggests that MR activation might induce genes important for migration of immune and stem cells to the maturing lung. Our data, therefore, suggest that in the normal lung, the actions of MR and GR while disparate, are complementary. Further study will be necessary to explore these effects of MR in the preterm lung.

## Acknowledgment

We wish to thank Nathan Jesse for assistance with the animal aspects of these experiments.

## Conflict of Interest

None declared.

## Appendix

**A1:** Genes differentially upregulated by aldosterone

ATP binding	34 genes, Adj *P*‐value = 1.59 × 10^−2^
GALK1	Galactokinase 1
CIT	Citron (rho‐interacting, serine/threonine kinase 21)
CHTF18	CTF18, chromosome transmission fidelity factor 18 homolog (*S. cerevisiae*)
SCYL1	SCY1‐like 1 (*S. cerevisiae*)
DDX19A	DEAD (Asp‐Glu‐Ala‐Asp) box polypeptide 19A
SMARCAL1	SWI/SNF related, matrix associated, actin dependent regulator of chromatin, subfamily a‐like 1
DAK	Dihydroxyacetone kinase 2 homolog (*S. cerevisiae*)
MCM7	Minichromosome maintenance complex component 7
TWF2	Twinfilin, actin‐binding protein, homolog 2 (*Drosophila*)
ABL1	c‐abl oncogene 1, non‐receptor tyrosine kinase
MCM4	Minichromosome maintenance complex component 4
ITPK1	Inositol‐tetrakisphosphate 1‐kinase
MCM5	Minichromosome maintenance complex component 5
MAST3	Microtubule associated serine/threonine kinase 3
ACTR5	ARP5 actin‐related protein 5 homolog (yeast)
HSP90AA1	Heat shock protein 90 kDa alpha (cytosolic), class A member 1
ACSM2B	Acyl‐CoA synthetase medium‐chain family member 2B
YARS	Tyrosyl‐tRNA synthetase
TRIP13	Thyroid hormone receptor interactor 13
MAP3K1	Mitogen‐activated protein kinase kinase kinase 1, E3 ubiquitin protein ligase
SEPHS1	Selenophosphate synthetase 1
TK1	Thymidine kinase 1, soluble
FBXO18	F‐box protein, helicase, 18
CCT3	Chaperonin containing TCP1, subunit 3 (gamma)
CDC6	Cell division cycle 6 homolog (*S. cerevisiae*)
MARS	Methionyl‐tRNA synthetase
ATP8B2	ATPase, aminophospholipid transporter, class I, type 8B, member 2
UBE2E1	Ubiquitin‐conjugating enzyme E2E 1
DHX32	DEAH (Asp‐Glu‐Ala‐His) box polypeptide 32
ABCB1	ATP‐binding cassette, sub‐family B (MDR/TAP), member 1
HK2	Hexokinase 2
HK1	Hexokinase 1
VPS4A	Vacuolar protein sorting 4 homolog A (*S. cerevisiae*)
CDK2	Cyclin‐dependent kinase 2

DNA repair genes	16 genes, Adj *P*‐value = 6.4 × 10^−3^
CHAF1B	Chromatin assembly factor 1, subunit B (p60)
BABAM1	BRISC and BRCA1 A complex member 1
INTS3	Integrator complex subunit 3
XRCC1	X‐ray repair complementing defective repair in Chinese hamster cells 1
CHAF1A	Chromatin assembly factor 1, subunit A (p150)
ABL1	c‐abl oncogene 1, non‐receptor tyrosine kinase
RBM14	RNA binding motif protein 14
ZSWIM7	Zinc finger, SWIM‐type containing 7
POLE	Polymerase (DNA directed), epsilon, catalytic subunit
TYMS	Thymidylate synthetase
ACTR5	ARP5 actin‐related protein 5 homolog (yeast)
CDK2	Cyclin‐dependent kinase 2
TRIP13	Thyroid hormone receptor interactor 13
FEN1	Flap structure‐specific endonuclease 1
DDB1	Damage‐specific DNA binding protein 1, 127 kDa
SSRP1	Structure specific recognition protein 1

Mitosis	15 genes, Adj *P*‐value = 6.4 × 10^−3^
RCC2	Regulator of chromosome condensation 2
CIT	Citron (rho‐interacting, serine/threonine kinase 21)
NCAPH	Non‐SMC condensin I complex, subunit H
CDC20	Cell division cycle 20 homolog (*S. cerevisiae*)
CDCA3	Cell division cycle associated 3
MYBL2	v‐myb myeloblastosis viral oncogene homolog (avian)‐like 2
CDC6	Cell division cycle 6 homolog (*S. cerevisiae*)
HAUS4	HAUS augmin‐like complex, subunit 4
RACGAP1	Rac GTPase activating protein 1
CENPM	Centromere protein M
CDC25B	Cell division cycle 25 homolog B (*S. pombe*)
CDK2	Cyclin‐dependent kinase 2
UBE2E1	Ubiquitin‐conjugating enzyme E2E 1
CKAP5	Cytoskeleton associated protein 5
E4F1	E4F transcription factor 1

Nuclear division	15 genes, Adj *P*‐value = 6.4 × 10^−3^
RCC2	Regulator of chromosome condensation 2
CIT	Citron (rho‐interacting, serine/threonine kinase 21)
NCAPH	Non‐SMC condensin I complex, subunit H
CDC20	Cell division cycle 20 homolog (*S. cerevisiae*)
CDCA3	Cell division cycle associated 3
MYBL2	v‐myb myeloblastosis viral oncogene homolog (avian)‐like 2
CDC6	Cell division cycle 6 homolog (*S. cerevisiae*)
HAUS4	HAUS augmin‐like complex, subunit 4
RACGAP1	Rac GTPase activating protein 1
CENPM	Centromere protein M
CDC25B	Cell division cycle 25 homolog B (*S. pombe*)
CDK2	Cyclin‐dependent kinase 2
UBE2E1	Ubiquitin‐conjugating enzyme E2E 1
CKAP5	Cytoskeleton associated protein 5
E4F1	E4F transcription factor 1

Cell cycle checkpoint	12 genes, Adj *P*‐value = 6.4 × 10^−3^
MCM4	Minichromosome maintenance complex component 4
PSMD13	Proteasome (prosome, macropain) 26S subunit, non‐ATPase, 13
MCM5	Minichromosome maintenance complex component 5
CDT1	Chromatin licensing and DNA replication factor 1
BABAM1	BRISC and BRCA1 A complex member 1
CDC20	Cell division cycle 20 homolog (*S. cerevisiae*)
INTS3	Integrator complex subunit 3
CDC6	Cell division cycle 6 homolog (*S. cerevisiae*)
MCM7	Minichromosome maintenance complex component 7
CDK2	Cyclin‐dependent kinase 2
DDB1	Damage‐specific DNA binding protein 1, 127 kDa
UBE2E1	Ubiquitin‐conjugating enzyme E2E 1

S phase of mitotic cell cycle	11 genes, Adj *P*‐value = 4.0 × 10^−4^
ABL1	c‐abl oncogene 1, non‐receptor tyrosine kinase
MCM4	Minichromosome maintenance complex component 4
POLE	Polymerase (DNA directed), epsilon, catalytic subunit
PSMD13	Proteasome (prosome, macropain) 26S subunit, non‐ATPase, 13
MCM5	Minichromosome maintenance complex component 5
CDT1	Chromatin licensing and DNA replication factor 1
CDC6	Cell division cycle 6 homolog (*S. cerevisiae*)
CDK2	Cyclin‐dependent kinase 2
MCM7	Minichromosome maintenance complex component 7
FEN1	Flap structure‐specific endonuclease 1
EZH2	Enhancer of zeste homolog 2 (*Drosophila*)

G1/S transition of mitotic cell cycle	10 genes, Adj *P*‐value = 2.03 × 10^−2^
MCM4	Minichromosome maintenance complex component 4
CDKN2C	Cyclin‐dependent kinase inhibitor 2C (p18, inhibits CDK4)
POLE	Polymerase (DNA directed), epsilon, catalytic subunit
PSMD13	Proteasome (prosome, macropain) 26S subunit, non‐ATPase, 13
MCM5	Minichromosome maintenance complex component 5
CDT1	Chromatin licensing and DNA replication factor 1
TYMS	Thymidylate synthetase
CDC6	Cell division cycle 6 homolog (*S. cerevisiae*)
CDK2	Cyclin‐dependent kinase 2
MCM7	Minichromosome maintenance complex component 7

G2/M transition of mitotic cell cycle	Eight genes, Adj *P*‐value = 2.57 × 10^−2^
TUBB4A	Tubulin, beta 4A class IVa
CDT1	Chromatin licensing and DNA replication factor 1
ABCB1	ATP‐binding cassette, sub‐family B (MDR/TAP), member 1
CDC6	Cell division cycle 6 homolog (*S. cerevisiae*)
CDC25B	Cell division cycle 25 homolog B (*S. pombe*)
HSP90AA1	Heat shock protein 90 kDa alpha (cytosolic), class A member 1
CDK2	Cyclin‐dependent kinase 2
CKAP5	Cytoskeleton associated protein 5

M/G1 transition of mitotic cell cycle	Eight genes, Adj *P*‐value = 1.1 × 10^−3^
MCM4	Minichromosome maintenance complex component 4
POLE	Polymerase (DNA directed), epsilon, catalytic subunit
PSMD13	Proteasome (prosome, macropain) 26S subunit, non‐ATPase, 13
MCM5	Minichromosome maintenance complex component 5
CDT1	Chromatin licensing and DNA replication factor 1
CDC6	Cell division cycle 6 homolog (*S. cerevisiae*)
CDK2	Cyclin‐dependent kinase 2
MCM7	Minichromosome maintenance complex component 7

Helicase activity	Seven genes, Adj *P*‐value = 3.26 × 10^−2^
MCM4	Minichromosome maintenance complex component 4
FBXO18	F‐box protein, helicase, 18
MCM7	Minichromosome maintenance complex component 7
MCM5	Minichromosome maintenance complex component 5
DDX19A	DEAD (Asp‐Glu‐Ala‐Asp) box polypeptide 19A
DHX32	DEAH (Asp‐Glu‐Ala‐His) box polypeptide 32
SMARCAL1	SWI/SNF related, matrix associated, actin dependent regulator of chromatin, subfamily a‐like 1

DNA replication initiation	Six genes, Adj *P*‐value = 3.0 × 10^−4^
MCM4	Minichromosome maintenance complex component 4
POLE	Polymerase (DNA directed), epsilon, catalytic subunit
MCM7	Minichromosome maintenance complex component 7
CDK2	Cyclin‐dependent kinase 2
MCM5	Minichromosome maintenance complex component 5
CDT1	Chromatin licensing and DNA replication factor 1

Histone‐lysine methyltransferase activity	Four genes, Adj *P*‐value = 2.18 × 10^−2^
WHSC1	Wolf‐Hirschhorn syndrome candidate 1
NSD1	Nuclear receptor binding SET domain protein 1
ASH2L	Ash2 (absent, small, or homeotic)‐like (*Drosophila*)
EZH2	Enhancer of zeste homolog 2 (*Drosophila*)

Positive regulation of cell cycle cytokinesis	Three genes, Adj *P*‐value = 2.03 × 10^−2^
RACGAP1	Rac GTPase activating protein 1
CDC6	Cell division cycle 6 homolog (*S. cerevisiae*)
CDC25B	Cell division cycle 25 homolog B (*S. pombe*)

DNA replication –dependent nucleosome assembly	Two genes, Adj *P*‐value = 9.9 × 10^−3^
CHAF1B	Chromatin assembly factor 1, subunit B (p60)
CHAF1A	Chromatin assembly factor 1, subunit A (p150)

Hexokinase activity	Two genes, Adj *P*‐value = 2.20 × 10^−2^
HK1	Hexokinase 1
HK2	Hexokinase 2

cAMP response element binding	Two genes, Adj *P*‐value = 2.69 × 10^−2^
CREB1	cAMP responsive element binding protein 1
E4F1	E4F transcription factor 1
